# We need a new acronym supplementing DAIR: Introducing DECRA

**DOI:** 10.1186/s42836-025-00328-9

**Published:** 2025-08-15

**Authors:** Edward J. McPherson, Rhidian Morgan Jones, Rami M. Sorial, Madhav Chowdhry

**Affiliations:** 1 Department of Orthopaedic Surgery, David Geffen School of Medicine, UCLA, Los Angeles, CA 90404 USA; 2 Essex and Suffolk Elective Orthopaedic Centre, Colchester Hospital, Turner Road, Colchester, CO4 5JL UK; 3 Department of Orthopaedics, Nepean Hospital, 60 A Derby Street, Penrith, NSW 2750 Australia; 4 Department of Orthopaedic Surgery, Jawaharlal Nehru Medical College, AMU, Aligarh, 202001 India; 5 Department of Continuing Education, University of Oxford, Oxford, OX1 2JA UK

## Abstract

The treatment for an acute Periprosthetic Joint Infection (PJI) is historically described using the acronym DAIR (Debridement, Antibiotics, Implant Retention). However, this acronym, by intention, does not imply that the modular parts of an implant system are always exchanged. There are many circumstances where modular exchange is not possible. It is well known that DAIR procedures with modular exchange show improved results. To reduce heterogeneity in the published literature, we introduce the supplementary acronym DECRA (Debridement, Modular Exchange, Component Retention, Antibiotics) for the treatment of acute PJI. The DECRA acronym will identify studies where modular exchange is always performed and will be separate from DAIR, where modular exchange is not performed or is unclarified. This will reduce heterogeneity and provide fidelity in research analysis. Moving forward, we advocate that the orthopaedic community, reviewers, and journal editors mindfully reflect this distinction in publications.

## Perspective

For acute periprosthetic joint infection (PJI), defined as early post-operative or late hematogenous infection of less than 4 weeks duration, treatment requires aggressive surgical intervention attempting to retain prosthetic implants and clear the infection [1–3]. The technique requires radical joint debridement, copious lavage, followed by a postoperative course of antimicrobial therapy.

The acronym ascribed to this treatment scheme is DAIR—Debridement, Antibiotics, Implant Retention, first introduced in 2009 [4]. However, the DAIR acronym, by original intention, does not mandate the exchange of modular bearings. This descriptor omission is relevant as studies show improved success when modular bearings are exchanged [5]. Moreover, for cases of revision implants and endoprostheses, the interfaces of connecting modular segments are also “hideout portals” for microbes, and all accessible modular segments require exchange. A more thorough debridement is possible when modular implants are removed, allowing comprehensive access to the joint. Further, modular exchange reduces total bioburden. Confusion was evident in the recent 3rd Meeting of the International Consensus Meeting for Musculoskeletal Infection, convened 8–10 May 2025, Istanbul, Turkiye (ICM-25). The authors attended this meeting, noting a contentious debate regarding consensus recommendations for DAIR treatment. Contention stemmed from a lack of clarity in differentiating study results (especially meta- and network analyses) based upon whether modular implants were exchanged. This stratification will remain keenly relevant as there are continued situations where modular bearings and segments are unable to be exchanged including: cases involving retired implant systems, financial restrictions prohibiting purchase of a modular bearing/segment (common in financially restricted healthcare systems), cases with an unrecognized implant system and inability to acquire modular parts for exchange in a timely fashion. These situations will always remain a challenge for optimizing acute PJI treatment. The inability to identify modular exchange in research articles creates confusion in assessing outcomes of DAIR, which came to a head at the ICM-25 meeting.

To provide clarity in literature, we advocate supplementing DAIR for acute PJI treatment with the acronym DECRA—Debridement, modular Exchange, Component Retention, Antibiotics [6]. In this way, manuscripts where all cases involve a DECRA procedure can be stratified from the more generic term DAIR, where cases of modular exchange may not be elaborated nor performed at all. We believe this would improve the objective evaluation of acute PJI treatment by easily identifying modular exchange cases in manuscript titles and keyword search lists. When standardized, DECRA would be a key descriptor for MeSH searches (Medical Subject Headings), improving categorization of search results. We advocate the adoption of the DECRA acronym for acute PJI treatment when all cases within a research manuscript include modular exchange. We recommend the DAIR acronym be used in the manuscript when: 1) the procedure did not involve modular bearing/segment exchange, 2) the manuscript does not identify whether the modular bearing/segment was exchanged or 3) there is a mixed cohort of DAIR and DECRA that is not separated for analysis (theoretically, this mixed group will have inferior results). In this way, the use of both DAIR/DECRA acronyms will provide greater fidelity in advanced data analysis.

Moving forward, we advocate that the orthopaedic community, reviewers, and journal editors mindfully reflect this distinction in publications. By reducing heterogeneity, we will be able to make more discerning comparisons in future advanced analysis, providing greater consensus. The DAIR/DECRA distinction highlights the necessity of everyone in orthopedic circles being on the same page.

## Conclusion

We advocate using the acronym DECRA as a supplement to DAIR. By stating a DECRA procedure, it is assured that all modular parts of an implant are exchanged. The DAIR acronym does not always imply modular exchange for many reasons. By using DECRA and DAIR acronyms in research articles, systematic review/meta-analyses for the treatment of acute PJI will have reduced heterogeneity and better clarity of analyzed outcome measures.

## Data Availability

No datasets were generated or analysed during the current study.
